# Mesenchymal Stem Cells Preconditioned with Hypoxia and Dexamethasone Promote Osteoblast Differentiation Under Stress Conditions

**DOI:** 10.7150/ijms.91222

**Published:** 2024-05-28

**Authors:** Miyako Shimasaki, Toru Ichiseki, Shusuke Ueda, Hiroaki Hirata, Norio Kawahara, Yoshimichi Ueda

**Affiliations:** 1 Department of Pathology 2, Kanazawa Medical University, Daigaku 1-1, Uchinada-machi, Kahoku-gun, Ishikawa 920-0293, Japan.; 2 Division of Translational Research, Department of Life Science, Medical Research Institute, Kanazawa Medical University, Daigaku 1-1, Uchinada-machi, Kahoku-gun, Ishikawa 920-0293, Japan.; 3 Department of Orthopaedic Surgery, Kanazawa Medical University, Daigaku 1-1, Uchinada-machi, Kahoku-gun, Ishikawa 920-0293, Japan.; 4 Department of Pathology, Keiju Medical Center, 94, Tomiokamachi, Nanao, Ishikawa 926-0816, Japan.

**Keywords:** mesenchymal stem cell, differentiation, dexamethasone, hypoxia, osteoblast

## Abstract

Bone marrow-derived mesenchymal stem cells (MSCs), which are capable of differentiating into osteoblasts, are used in effective regenerative therapies. MSCs must be prompted to differentiate into osteoblasts for MSC transplantation to be effective. In this study, osteoblast differentiation markers involved in bone formation were evaluated to investigate the stress resistance of bone marrow-derived rat MSCs to dexamethasone and hypoxia and their ability to differentiate into osteoblasts. MSCs were allowed to differentiate into osteoblasts for 21 days in three different environments (dexamethasone treatment, hypoxic conditions [1% oxygen], or both). Osteoblast differentiation potential was evaluated according to alkaline phosphatase levels and a mineralisation assay. Immunofluorescence staining was used to determine the protein expression of the osteoblast differentiation markers type I collagen and osteopontin. MSCs differentiated into osteoblasts under hypoxic conditions but differentiated more slowly upon treatment with dexamethasone and dexamethasone plus hypoxia relative to the control. MSCs preconditioned with dexamethasone or hypoxia and then allowed to differentiate into osteoblasts under similar conditions differentiated comparably to control MSCs. MSCs that developed resistance to dexamethasone or hypoxia differentiated more quickly into osteoblasts than those that did not. The findings suggest that increasing the resistance of MSCs to stress by preconditioning them via dexamethasone or hypoxia exposure could result in more rapid differentiation into osteoblasts following transplantation.

## Introduction

The capacity of bone marrow mesenchymal stem cells (MSCs) to self-renew and differentiate into various tissues has prompted investigations into their potential clinical applications. In orthopaedic surgery, substantial research involving the anti-inflammatory and immunosuppressive effects of MSCs is underway 1. Recent promising results reported in scaffold-based trials on cartilage regeneration suggest that the clinical application of MSCs is nearing realisation. Other studies of MSCs in post-fracture healing and osteoporosis treatment have heightened expectations of clinical applications in these areas.

Glucocorticoids, which offer excellent therapeutic efficacy, are widely used to treat a variety of conditions. This class of drugs, however, damages bones, causing osteoporosis and refractory osteonecrosis. Therefore, ways to prevent and treat these adverse reactions must be established to use glucocorticoids safely in the clinic. A recent study reported the effect of MSCs in preventing refractory glucocorticoid-associated osteonecrosis in an animal model 2. Glucocorticoid-associated osteonecrosis is partially caused by bone hypoxia and the cytotoxic effects of glucocorticoids in tissues 3-5. Advanced osteonecrosis is commonly treated using joint replacement or other surgical procedures. Therefore, an effective therapy that can be administered in the stages of osteonecrosis before joint replacement is necessary as it would reduce the invasiveness of treatment. Another concern is the increased risk of fractures due to glucocorticoid-induced osteoporosis in long-term users. Moreover, treatments for osteoporosis are also needed to improve the activities associated with daily living in patients using glucocorticoids. The efficacy of MSCs in treating age-related osteoporosis indicates that they could also be beneficial in treating glucocorticoid-induced osteoporosis 6,7.

Injected or transplanted MSCs for treating glucocorticoid-associated osteonecrosis or glucocorticoid-induced osteoporosis must function well under hypoxic conditions in the bone and glucocorticoid-exposed tissues. The interior of all bones is hypoxic, even in healthy individuals. In the presence of glucocorticoids, the bone interior becomes ischaemic or more hypoxic, inducing glucocorticoid-associated cytotoxicity, contributing to osteonecrosis. To better clarify the processes involved in this phenomenon, recent studies have investigated osteonecrosis by recreating hypoxia- and glucocorticoid-stressed environments *in vitro* 4,8,9. In the present study, we investigated the ability of MSCs to differentiate into bone *in vitro* in glucocorticoid- and hypoxia-stressed environments, which could cause osteonecrosis in the human body. We also cultured MSCs preconditioned in these environments to determine if exposure to these stresses would enhance their functional resistance.

## Materials and Methods

### Cell culture

Rat MSCs derived from the bone marrow (Cyagen, Silicon Valley, CA, USA) were maintained in a stem cell growth medium (Cyagen). Cell lines were cultured at 37°C under 20% O_2_ and 5% CO_2_. The cell monolayers reached 80% confluency after incubation for 48 h. MSCs were then subjected to three different conditions for 72 h: exposure to dexamethasone (MSD, Tokyo, Japan) at a concentration of 0.4 µg/ml (Dex group), hypoxia at a 1% oxygen concentration (Hypoxia group), or both (Dex/Hypoxia group). As a control, MSCs were cultured under 20% oxygen in a culture medium without dexamethasone. These three groups of MSCs (Dex, Hypoxia, and Dex/Hypoxia groups) were then differentiated into osteoblasts. After exposing MSCs to the corresponding treatments/conditions for 72 h, the spent medium was replaced with an osteoblast differentiation medium. Finally, the cells were differentiated into osteoblasts under the same stressors for 1, 7, and 21 days.

### Cell viability assay

Cell viability assays were performed using a Cell Meter™ Apoptotic and Necrotic Multiplexing Detection Kit (AAT Bioquest, Pleasanton, CA, USA) according to the manufacturer's instructions. The percentages of apoptotic/necrotic cells relative to the total cell number were determined. Apoptotic cells were detected by staining with fluorescein-labeled Apopxin™ Green (green fluorescence). Necrotic cells were detected via staining with 7-AAD, a highly positively charged nucleic acid probe; it is impermeant to live cells and early apoptotic cells but stains necrotic cells and late apoptotic cells (entering into secondary necrosis) with red fluorescence. Viable cells were stained blue by CytoCalcein™ Violet 450. Fluorescence-positive cells were evaluated via phase contrast and fluorescence (470 nm and 530 nm LED modules) microscopy using a BZ-X700 microscope (Keyence, Tokyo, Japan).

### Determination of cell proliferation

Cell proliferation analysis was performed at 24, 48, 72, and 96 h of exposing rat MSCs to dexamethasone. Viable cells were identified using a Cell Counting Kit-8 (CCK-8; Dojindo Laboratories, Kumamoto, Japan) assay. The spectrophotometric absorbance was detected on an iMark microplate reader (Bio-Rad Laboratories, Inc, Hercules, CA, USA) at each time point.

### Alkaline phosphatase (ALP) staining

The osteoblastic differentiation potency of the rat MSCs was compared over time using ALP staining 10,11. Briefly, the rat MSCs were cultured in a 24-well plate in an osteoblast differentiation medium. On days 1, 7, and 21 of differentiation, histochemical staining for ALP activity was conducted using a tartrate-resistant acid phosphatase and ALP double-stain kit (Takara Biomedical, Shiga, Japan). For ALP staining, the cells were washed with phosphate-buffered saline (PBS) twice, and then 250 μl of the fixation solution was added to each well for 5 min. After washing with sterile distilled water, 250 μl of the ALP substrate solution was added to each well and allowed to react for 45 min at 37ºC to stain the cells. All samples were then scanned (ESPER-SCANNER ES-2000, EPSON, Tokyo, Japan), and ALP intensity was measured using the Multi Gauge software package (v3.1; Fujifilm, Tokyo, Japan).

### Immunostaining for collagen I, osteopontin, Runx2, and ATF4

To study the effect of dexamethasone or hypoxia on the osteoblastic differentiation potential of MSCs, we performed immunocytochemical staining of osteoblast differentiation markers (collagen I and osteopontin) and osteoblast differentiation transcription factors (Runx2 and ATF4) 12. Four groups of MSCs (Dex, Hypoxia, Dex/Hypoxia, and the control group) were used to generate osteoblast-differentiated cell populations for 1, 7, or 21 days. Cultured cells were fixed in 4% paraformaldehyde, washed in PBS, and permeabilised with 0.3% Triton X-100 in PBS. Nonspecific binding was blocked by incubating the cells with 10% bovine serum albumin (Dako Cytomation, Santa Clara, CA, USA) in PBS for 15 min. The cells were then incubated with anti-collagen-I (Arigo Biolaboratories, Hsinchu City, Taiwan), anti-osteopontin (Abcam, Cambridge, UK), anti-Runx2 (Abcam), and anti-ATF4 (Cell Signaling Technology, Danvers, MA, USA) antibodies for 2 h at concentrations of 1.0, 2.0, 2.0, and 10.0 mg/ml, respectively. Next, the cells were incubated with the corresponding fluorescent Alexa 488-labeled secondary antibodies (Thermo Fisher Scientific, Waltham, MA, USA) and then with DAPI for 30 min. After washing, Prolong™ Diamond Antifade Mountant (Thermo Fisher Scientific) was added to the cells, followed by mounting on glass slides and covering with coverslips. Finally, images were taken using a confocal microscope (Zeiss-LSM710, Zeiss, Baden-Württemberg, Germany).

### Statistical analysis

All data are presented as the means ± standard deviation (SD). Differences between groups were assessed using a one-way analysis of variance followed by Fisher's protected least significant difference post hoc test. Differences were considered significant at *p* < 0.05.

## Results

### Effects of dexamethasone on MSCs

**Figure 1:**
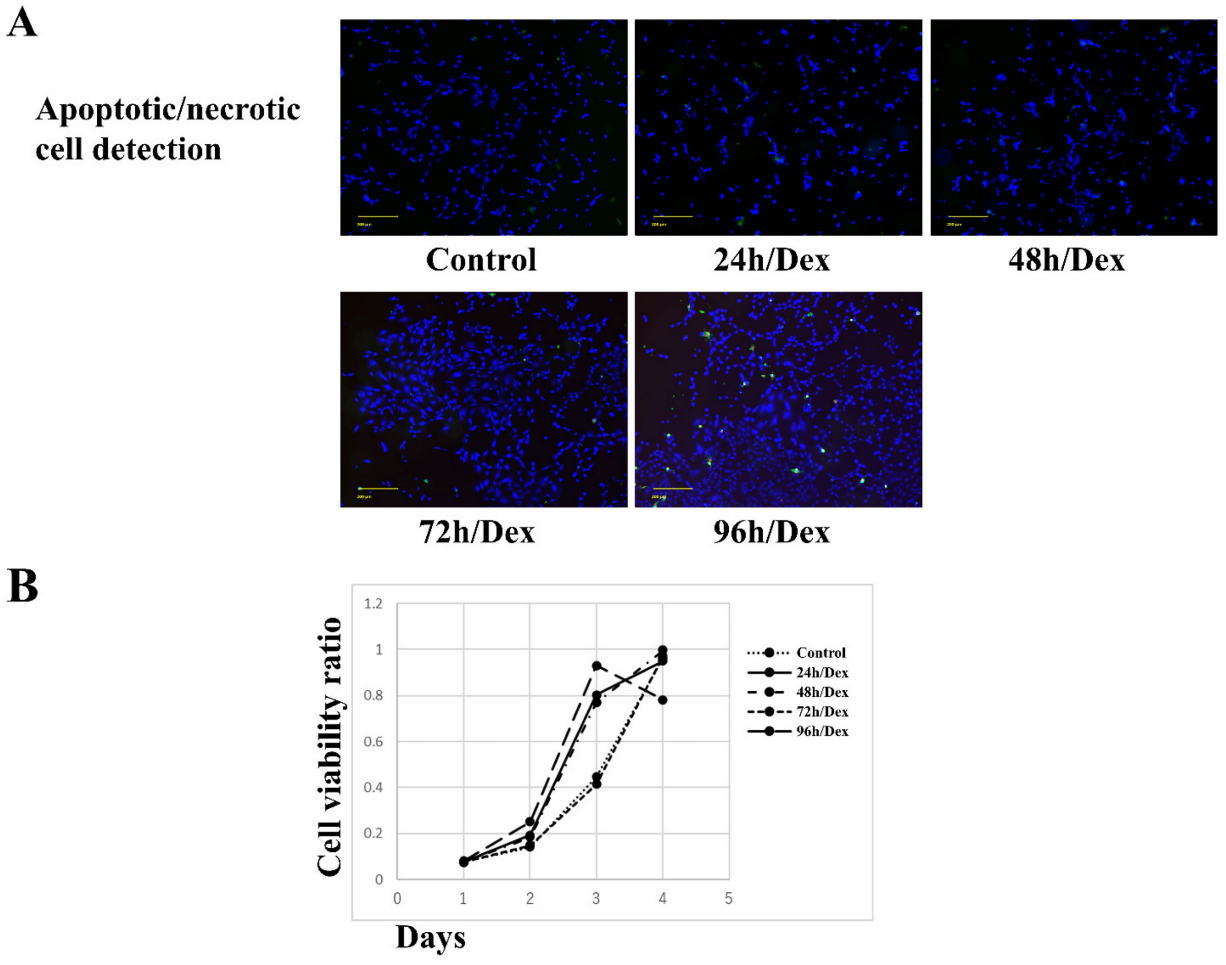
** Effects of dexamethasone treatment on the apoptosis/necrosis and proliferation of rat bone marrow mesenchymal stem cells (MSCs).** (A) MSC apoptosis/necrosis. Shown are fluorescence images of live (blue), apoptotic (green), and necrotic (red) MSCs after treatment with dexamethasone for 24, 48, 72, or 96 h (24h/Dex, 48h/Dex, 72h/Dex, and 96h/Dex, respectively). Scale bar: 200 µm. (B) Cell Counting Kit-8 assay results after treating MSCs with dexamethasone for 24, 48, 72, or 96 hours (24 hours/Dex, 48 hours/Dex, 72 hours/Dex, and 96 hours, respectively). Exposing MSCs to dexamethasone for up to 72 h had no effect on cell apoptosis/necrosis, survival, and proliferation.

No apparent apoptosis or necrosis was observed in MSCs exposed to dexamethasone for 24, 48, or 72 h. However, apoptosis and necrosis were observed in MSCs exposed to dexamethasone for 96 h (Figure 1A). MSCs exposed to dexamethasone for 24, 48, or 72 h maintained cell proliferation comparable to the control. Similarly, MSCs exposed to dexamethasone for 96 h showed decreased cell proliferation compared to the control (Figure 1B). These results suggest that dexamethasone treatment for up to 72 h did not affect MSCs.

### Effects of exposure to dexamethasone or hypoxia on the potential of rat bone marrow-derived MSCs to differentiate into osteoblasts

#### ALP-based evaluation of bone formation

**Figure 2:**
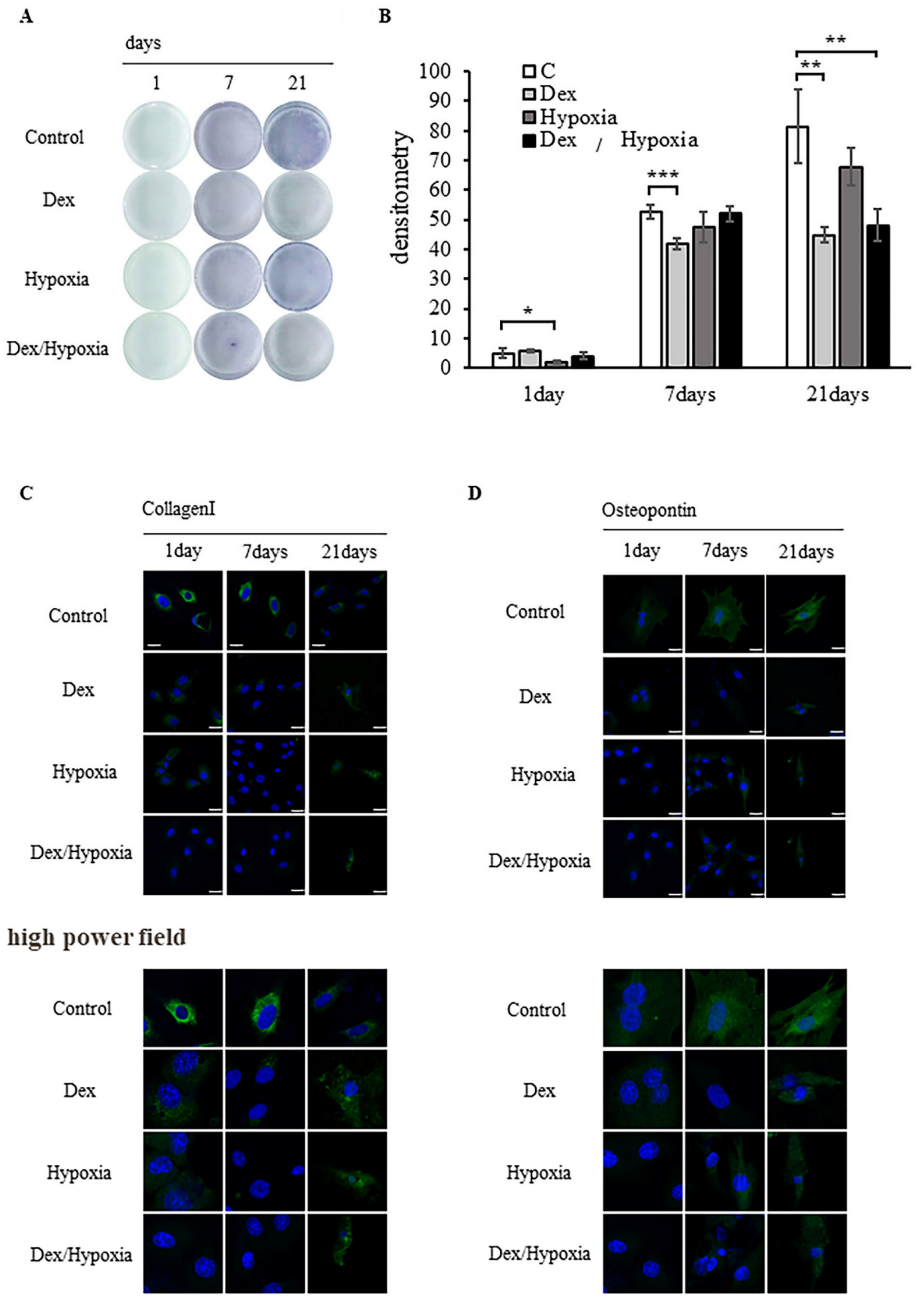
** Effect of rat bone marrow-derived mesenchymal stem cells (MSCs) on osteoblast differentiation in cytotoxic environments.** (A) Osteogenic differentiation was assessed by determining alkaline phosphatase (ALP) activity after 21 days of culture in an osteogenic medium. (B) Quantitative analysis of ALP activity. The ALP activity of MSCs differentiated into osteoblasts under stress decreased compared to the control (* *p* < 0.05, ** *p* < 0.01, and *** *p* < 0.001). (C, D) Expression of type I collagen and osteopontin in MSCs after 1, 7, or 21 days of exposure to cytotoxic stress. Shown are representative immunofluorescence images for DAPI (blue), type I collagen (C, green), or osteopontin (D, green) after exposing cells to dexamethasone (Dex), hypoxia (Hypoxia), or both dexamethasone and hypoxia (Dex/Hypoxia). Scale bar = 20 μm.

We investigated the potential of MSCs to differentiate into osteoblasts when exposed to dexamethasone or hypoxia. The progression of bone formation was evaluated by detecting the osteoblast differentiation marker ALP. In the control group, ALP activity increased over time, from days 1 to 21 of osteoblast differentiation. In each exposure group, differentiation was comparable to the control on days 1 and 7, but became slower than the control on day 21. Differentiation was particularly delayed in the MSCs exposed to Dex and Dex/hypoxia (* *p* < 0.05, ** *p* < 0.01, *** *p* < 0.001) (Figures 2A, 2B).

#### Evaluation of osteoblast differentiation using type I collagen and osteopontin

The expression of the early differentiation marker type I collagen was higher than in the control on days 1 and 7 of differentiation, but was lower than the control on day 21. Osteopontin expression in the control increased from days 1 to 21 of osteoblast differentiation. Type I collagen and osteopontin expression were lower in each group of exposed MSCs than in the control. Although type I collagen and osteopontin were expressed on day 21 in each group of exposed MSCs, their nuclei were markedly smaller than the nuclei of the control. Similar results were confirmed in high power fields (Figures 2C, 2D). The expressions of Runx2 and ATF4 are shown in Figure S1.

### Effects of preconditioning of rat bone marrow-derived MSCs to dexamethasone or hypoxia treatment on osteoblast differentiation

#### ALP-based evaluation of bone formation

**Figure 3:**
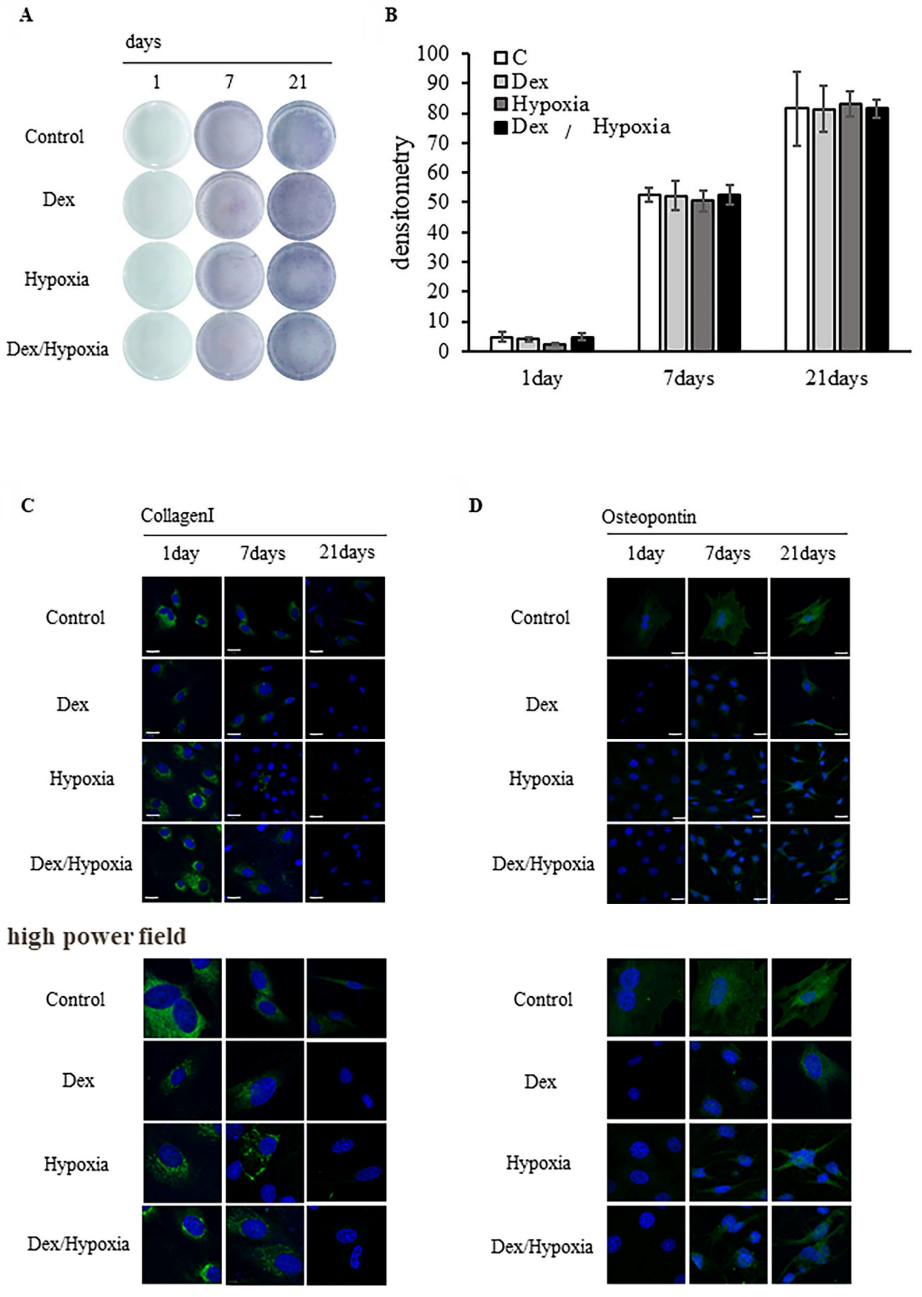
** Effect of dexamethasone or hypoxia on the osteoblast differentiation of rat bone marrow-derived mesenchymal stem cells (MSCs) under 72 h of preconditioning exposure to stress conditions.** (A) Osteogenic differentiation was assessed by determining alkaline phosphatase (ALP) activity after 21 days of culture in an osteogenic medium. (B) Quantitative analysis of the ALP activity in MSCs. The ALP activity of MSCs preconditioned with stress conditions for 72 h before differentiation was comparable to the controls. (C, D) Expression of type I collagen and osteopontin in MSCs after 1, 7, or 21 days of exposure to cytotoxic stress. Shown are representative immunofluorescence images for DAPI (blue), type I collagen (C, green), or osteopontin (D, green) after exposing cells to dexamethasone (Dex), hypoxia (Hypoxia), or both dexamethasone and hypoxia (Dex/Hypoxia). Scale bar = 20 μm.

MSCs preconditioned with dexamethasone or hypoxia treatment were allowed to differentiate into osteoblasts under identical exposure conditions. ALP activity increased over time from days 1 to 21 of differentiation in all exposure groups and the controls; osteoblast differentiation in the exposure groups was comparable to that in the control (Figure 3A). Osteoblast differentiation did not differ significantly among the Dex, hypoxia, Dex/hypoxia, and control groups (Figure 3B).

#### Evaluation of osteoblast differentiation using type I collagen and osteopontin

Type 1 collagen was expressed on day 1 of differentiation from MSCs into osteoblasts in all preconditioned groups, including the control, and was not expressed on day 21 (Figure 3C). In addition, osteopontin was expressed on days 7 and 21 of differentiation in all groups, including the control (Figure 3D). On day 21 of osteoblast differentiation, the morphology of cells and nuclei in all preconditioned groups remained comparable to the control. Similar results were confirmed even in the high power fields.

## Discussion

Since inducing the differentiation of transplanted bone marrow-derived MSCs into osteoblasts could provide a therapeutic effect against glucocorticoid-induced osteoporosis and glucocorticoid-associated osteonecrosis, we investigated how well MSCs differentiate into osteoblasts exposed to dexamethasone, hypoxia, or both. MSCs exposed to hypoxia differentiated into osteoblasts in a manner comparable to those grown in a normoxic environment. However, MSCs differentiated into osteoblasts more slowly when exposed to dexamethasone alone or dexamethasone and hypoxia. These findings suggest that MSCs transplanted into patients treated with dexamethasone or dexamethasone in the hypoxic environment of the bone (which are risk factors for osteoporosis or osteonecrosis) could lose their potential to differentiate and thus fail to provide a sufficient therapeutic effect.

Generally, previous studies showed that MSCs preconditioned in an environment identical to that of the transplantation site, such as MSCs preconditioned in a hypoxic environment for transplantation into a patient with ischaemic disease, produce a stronger therapeutic response 13,14. These studies also indicate that MSCs preconditioned in an environment similar to their transplantation site are more resistant to the stresses prevalent at such sites and could provide a sufficient therapeutic effect. Studies that investigated the effects of MSC preconditioning in patients with ischaemic disease found that preconditioning conferred stress resistance and enhanced treatment efficacy 15-18. MSCs have also been reported to be highly resistant to stressful environments 19. Our results showed that preconditioning for up to 72 h did not affect the cell proliferation ability of MSCs after exposing them to dexamethasone. We then hypothesised that increasing the resistance of MSCs to stress could allow transplanted MSCs to sufficiently differentiate into osteoblasts. To verify this hypothesis, we preconditioned MSCs for 72 h with dexamethasone, a hypoxic environment with 1% oxygen, or both, and then allowed the cells to differentiate into osteoblasts. When cultured under stressed conditions, the preconditioned MSCs differentiated comparably to MSCs cultured under normoxic conditions. This finding indicates that preconditioning MSCs to environmental stress increases their resistance to stress. In other words, MSCs preconditioned in an environment similar to the stressed environment present in the site of transplantation may enhance MSC survival at that site. Although further verification is needed using *in vivo* animal models of stress resistance, the findings of our study appear promising.

## Supplementary Material

Supplementary figure.

## Abbreviations

ALP: alkaline phosphatase
MSCs: mesenchymal stem cells
PBS: phosphate-buffered saline
