# Supplementary material for: Mesenchymal Stem Cells Preconditioned with Hypoxia and Dexamethasone Promote Osteoblast Differentiation Under Stress Conditions
Source: Int J Med Sci. 2024 May 28;21(8):1511–7. doi: 10.7150/ijms.91222 (PMC11186427; doi:10.7150/ijms.91222)
Supplement: Supplementary file 1 — Supplementary figure. [file ijmsv21p1511s1.pdf]

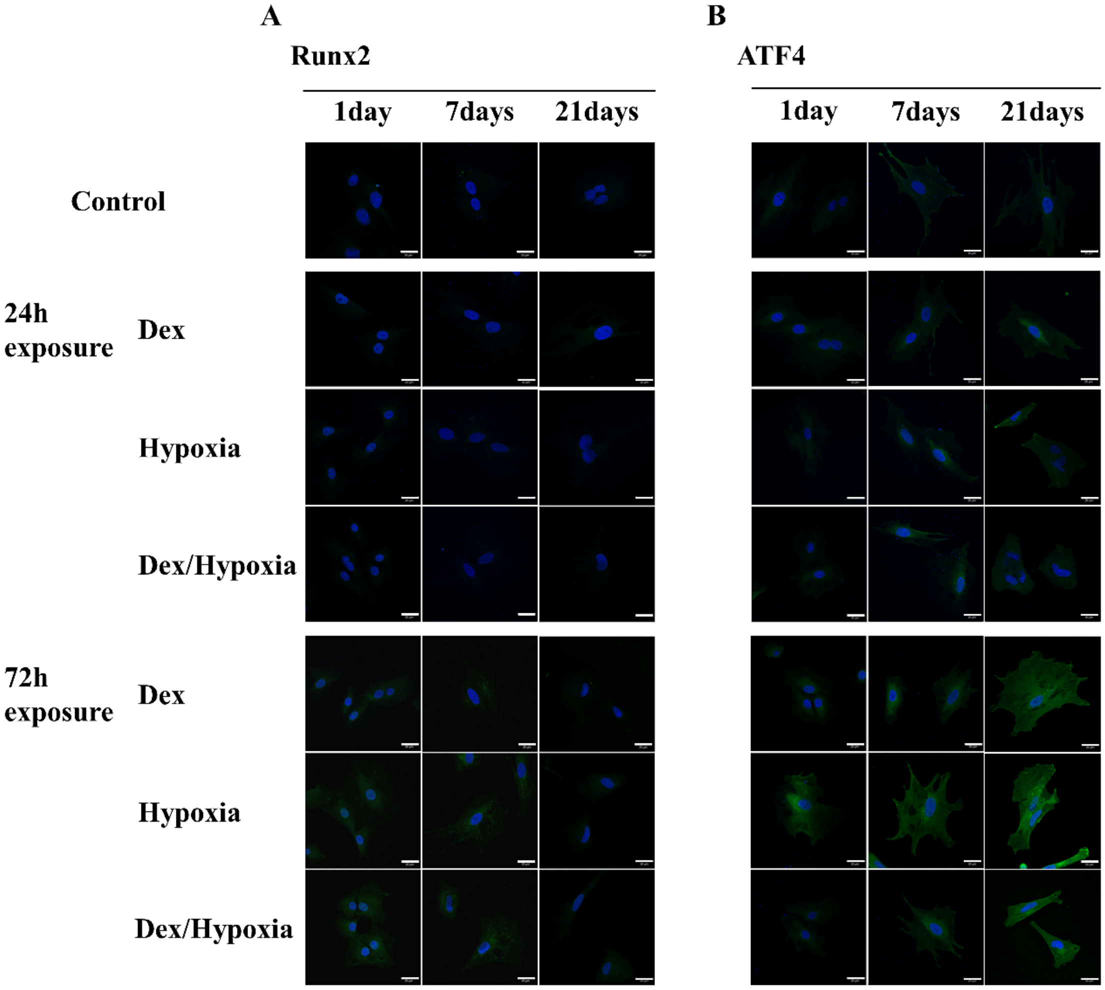

Figure S1. Expression of Runx2 and ATF4, transcription factors involved in bone formation, in MSCs induced to differentiate into osteoblasts after exposure to cytotoxic stress. (A) Immunofluorescence images showing Runx2 expression (green) and DAPI staining (blue) in MSCs subjected to cytotoxic stress due to dexamethasone, hypoxia, or both. Scale bars = 20 μm. No significant difference in Runx2 expression was observed in MSCs exposed for 24 h compared to the controls. However, MSCs exposed for 72 h showed higher Runx2 expression on the first day of differentiation than the control. (B) Representative immunofluorescence images of ATF4 (green) and DAPI staining (blue) in MSCs exposed to dexamethasone (Dex), hypoxia (Hypoxia), or both dexamethasone and hypoxia (Dex/Hypoxia). Scale bars = 20 μm. In all groups, ATF4 (which

contributes to bone mineralization) was weakly expressed on day 1 and expressed at comparably high levels across groups on days 7 and 21. ATF4 expression in MSCs preconditioned for 24 h did not differ much from that in the control. However, ATF4 expression in MSCs preconditioned for 72 h was markedly higher than in the control.
